# LncRNA H19 alleviates sepsis-induced acute lung injury by regulating the miR-107/TGFBR3 axis

**DOI:** 10.1186/s12890-022-02091-y

**Published:** 2022-09-30

**Authors:** Xiuling Hao, Huiqiang Wei

**Affiliations:** Department of Respiratory Medicine, East Hospital, The Second Hospital of Hebei Medical University, No. 80, Huanghe Avenue, East Development Zone, Shijiazhuang City, 050000 Hebei Province People’s Republic of China

**Keywords:** Sepsis, Acute lung injury, LncRNA H19, miR-107, TGFBR3, Apoptosis, Inflammatory cytokines, Pulmonary microvascular endothelial cells

## Abstract

**Objective:**

Acute lung injury (ALI) increases sepsis morbidity and mortality. LncRNA H19 plays a critical role in sepsis. miR-107 is highly-expressed and TGFβ type III receptor (TGFBR3) is poorly-expressed in sepsis, yet their roles in sepsis development require further investigation. This study aimed to investigate the mechanism of H19 in alleviating sepsis-induced ALI through the miR-107/TGFBR3 axis.

**Methods:**

Mice were intravenously injected with Ad-H19 adenovirus vector or control vector one week before establishing the mouse model of cecal ligation and puncture (CLP). Pulmonary microvascular endothelial cells (PMVECs) were transfected with oe-H19 or oe-NC plasmids and then stimulated by lipopolysaccharide (LPS). Lung injury was assessed via hematoxylin–eosin staining, measurement of wet-to-dry (W/D) ratio, and TUNEL staining. Levels of H19, miR-107, and TGFBR3 were determined by RT-qPCR. Apoptosis of PMVECs was evaluated by flow cytometry. Levels of Bax and Bcl-2 in lung tissues and PMVECs were measured using Western blot. Total protein concentration and the number of total cells, neutrophils, and macrophages in bronchoalveolar lavage fluid (BALF) were quantified. Levels of TNF-α, IL-1β, IL-6, and IL-10 in BALF, lung tissues, and PMVECs were measured by ELISA. Cross-linking relationships among H19, miR-107 and TGFBR3 were verified by dual-luciferase and RIP assays.

**Results:**

H19 was poorly-expressed in CLP-operated mice. H19 overexpression attenuated sepsis-induced ALI, which was manifested with complete alveolar structure, decreased lung injury score and lung W/D ratio, and inhibited apoptosis in CLP-operated mice, which was manifested with decreased number of TUNEL-positive cells and Bax level and increased Bcl-2 level. CLP-operated mice had increased concentration of total protein and number of total cells, neutrophils, and macrophages in BALF, which was nullified by H19 overexpression. H19 overexpression declined levels of TNF-α, IL-1β, and IL-6 and elevated IL-10 levels. H19 inhibited LPS-induced PMVEC apoptosis and pro-inflammatory cytokine production. H19 targeted TGFBR3 as the ceRNA of miR-107. miR-107 overexpression or silencing TGFBR3 partially averted the inhibition of H19 overexpression on LPS-induced PMVEC apoptosis and pro-inflammatory cytokine production.

**Conclusion:**

LncRNA H19 inhibited LPS-induced PMVEC apoptosis and pro-inflammatory cytokine production and attenuated sepsis-induced ALI by targeting TGFBR3 as the ceRNA of miR-107.

**Supplementary Information:**

The online version contains supplementary material available at 10.1186/s12890-022-02091-y.

## Introduction

Sepsis refers to a life-threatening organ dysfunction attributed to the dysregulated host response to infection [1–4], which may lead to long-term cognitive disorder [5]. As a common systemic inflammatory response syndrome (SIRS) [6–8], sepsis results in multiple organ damage, and the lung is the most susceptible to sepsis [9, 10], and that is when acute lung injury (ALI) occurs, the consequences of which include hypoxemic respiratory failure and even a life-threatening condition [11]. Given the complexity of its pathogenesis, further clarification of the pathogenesis would be instrumental to the early diagnosis, assessment of efficacy, and prognosis prediction of sepsis-induced ALI [12].

Long non-coding RNAs (LncRNAs), in its essence, are a class of common RNA molecules in eukaryotic cells with approximately 200 nucleotides and the property of regulating gene expression, and there has been renewed interest in their effect on human diseases in recent years [13, 14]. Multiple lncRNAs can mitigate inflammatory reactions by mediating inflammatory cytokines [15]. Recently, considerable literature has grown up around the theme of lncRNAs and sepsis-associated diseases: (1) lncRNA CASC2 attenuates sepsis-induced injury in pulmonary alveolar epithelial cells via regulating the microRNA (miR)-152-3p/PDK4 axis [16]; (2) lncRNA XIST accelerates inflammatory responses and apoptosis in lipopolysaccharide (LPS)-induced ALI [17]; (3) knockdown of lncRNA MALAT1 ameliorates ALI via apoptosis inhibition [11]. LncRNA H19 proved a promising regulator of miRs [18] that is abundantly expressed in endothelial cells [19]. Recent evidence suggests that H19 plays a regulatory role in mouse pulmonary microvascular endothelial cells (PMVECs) [20]. Meanwhile, H19 is recognized as an inhibitory factor of sepsis-induced kidney injury and ALI [21, 22]. It has previously been observed that H19 is downregulated in mice with LPS-induced sepsis [23] and in sera of sepsis patients [24]. Additionally, existing research recognizes the correlation between H19 and inflammatory reaction and oxidative stress in rats with sepsis-triggered ALI [22]. However, the previous studies of lncRNA H19 have not dealt with the action and concrete mechanism of H19 in sepsis-evoked ALI.

miRNAs are construed as endogenous and highly conserved non-coding RNA molecules with a length of about 18–24 nucleotides [12, 25]. miRNAs are capable of regulating the expression patterns of target genes and participating in various cellular activities through binding to the specific sites of mRNA [26]. Importantly, miRNAs play essential regulatory roles in sepsis [27]. In patients with sepsis-induced acute kidney injury, miR-107 could manipulate the production of tumor necrosis factor-α (TNF-α) in endothelial cells to induce tubular cell injury [28]. Additionally, miR-107 is strongly-expressed in the whole blood of mice with LPS-induced sepsis [29] or mice with pulmonary ischemia/reperfusion injury [30]. Nevertheless, few writers have been able to draw on any systematic research into its role in sepsis progression. In addition, the TGFβ type III receptor (TGFBR3) is an ALI-related gene [31] and an important gene that is downregulated in sepsis [32]. Inhibition of TGFBR3 can aggravate inflammation by activating the TGF-β pathway [33]. On the other hand, H19 can downregulate miR-107 and thus relieve neural stem cell injury provoked by hypoxia [34]. According to the predicted results on Starbase bioinformatics software, a potential binding site exists between H19 and miR-107, and TGFBR3 is a known downstream target of miR-107. Hence, we hypothesized that lncRNA H19 limited inflammatory responses in sepsis-induced ALI through the miR-107/TGFBR3 axis. The current study attempted to prove the hypothesis with the expectation to understand the mechanism of H19 in sepsis, offer novel therapeutic targets, and lay a theoretical foundation for the treatment of sepsis.

## Material and methods

### Ethics statement

The animal experiments were carried out in the light of the guidelines for the Care and Use of Laboratory Animals in research and got the approval of the Laboratory Animal Welfare and Ethics Committee of The Second Hospital of Hebei Medical University. Every effort was made to minimize the number of animals used and their pain. All methods are reported under ARRIVE guidelines.

### Establishment of the mouse model of sepsis-induced ALI

Healthy adult male C57BL/6 mice aged 8–9 weeks were bought from the Vital River Laboratory Animal Technology [SCXK (Beijing) 2016–0008, Beijing, China] and placed in a controlled environment at 22–24 °C with 60% humidity under a 12-h light/dark cycle. All mice had free access to food and water. After one-week acclimatization, cecal ligation and puncture (CLP) was performed to establish the mouse model using a 22-gauge needle. After anesthesia with 40 mg/kg pentobarbital sodium [2% (w/v)], the abdominal hair was removed and the surface of the abdominal skin was sterilized with iodine, and then a 3-cm incision was made through the midline of the anterior abdomen to remove the cecum with special attention not to cause damage to the mesentery or destroy the integrity of the intestinal wall. The cecum was ligated using surgical sutures at 1.5 cm from the end and subsequently the cecum was perforated once at the distal end of the ligation. After squeezing a small amount of feces from the perforation area, the ligated cecum was placed back in the peritoneal cavity and the abdominal incision was closed layer by layer. Normal saline was administered in mice by subcutaneous injection every 6-h for fluid resuscitation immediately after surgery [35]. CLP surgery on each mouse was finished within 10 min. Mice in the sham group were operated on with the same procedures of laparotomy without CLP.

### Animal grouping and treatment

Totally 48 mice were arbitrarily divided into 4 groups (N = 12): sham group, CLP group, CLP + Ad-GFP group, and CLP + Ad-H19 group. One week prior to the surgery, Gateway LR Clonase II (Invitrogen, Carlsbad, CA, USA) was used to transfer H19 cDNA [(or its negative control (NC)] to the adenovirus vector [36], and mice in the CLP + Ad-GFP group and CLP + Ad-H19 group eceived an intravenous injection of 20 μL Ad-H19 or Ad-GFP adenovirus vectors (10^7^ particles/μL) via the caudal vein [37]. The adenovirus vectors overexpressing H19 and carrying enhanced green fluorescent protein gene (Ad-H19) and its control (Ad-GFP) were obtained from Life Technologies (Shanghai, China). Mice were returned to the cages with free access to water and food after CLP and fed for 1 week before subsequent experimentation. After mice were euthanized using 150 mg/kg pentobarbital sodium, bronchoalveolar lavage fluid (BALF) was immediately collected from 12 mice in each group [37, 38], and the right lungs were collected from 6 of these mice for TUNEL staining and hematoxylin–eosin (H&E) staining, and the left lungs for preparation of tissue homogenate, and the lung tissues of the remaining 6 mice were used for measurement of wet-to-dry (W/D) ratio.

### H&E (Hematoxylin–Eosin) staining

The fresh lung tissues of mice (right upper lobe) were fixed with 10% paraformaldehyde (PFA, Sigma-Aldrich, St. Louis, MO, USA), dehydrated with ethanol of gradient concentrations and xylene, embedded in paraffin, and sliced at 5 μm using a slicer (Leica RM2145, Leica, Wetzlar, Germany). After dewaxing, the slices were stained with H&E, sealed with neutral gum, and observed under an optical microscope to evaluate pathological changes. As previously mentioned [39], lung injury was assessed in a blinded fashion using the lung injury scoring system recommended by the American Thoracic Society. In brief, alveolar congestion, alveolar wall thickening and edema, and inflammatory cell infiltration were scored from 1 to 3 (0: none; 1, mild; 2, moderate; 3, severe) with a total score of 9 [38].

### Measurement of lung W/D ratio

Lung W/D ratio reflected the lung water content and could be used to evaluate pulmonary edema. Following euthanasia, the fresh right upper lobe was isolated and weighed (wet weight) after removing the exudation and residual blood on the surface using the filter paper, dried in an oven for 24-h at 180 °C, and weighed (dry weight). The W/D ratio was subsequently calculated.

### Cell counting and protein concentration quantification

BALF was centrifuged for 10 min at 4 °C and 1500 ×g. The precipitated cells were resuspended again in PBS buffer. Cells were observed by Wright-Giemsa staining (Sigma-Aldrich) and counted in a double-blind method. The concentration of protein in the supernatant was measured using the bicinchoninic acid (BCA) protein detection method (Pierce, Rockford, IL, USA).

### Terminal deoxyribonucleotidyl transferse (TdT)-mediated biotin-16-dUTP nick-end labelling (TUNEL) staining

Cell apoptosis was assessed using TUNEL and 4′,6-diamidino-2-phenylindole (DAPI) staining solution (Thermo Fisher Scientific, Waltham, MA, USA). To distinguish apoptotic cells and non-apoptotic cells, DAPI was used to stain the nuclei, and apoptotic cells were counted by TUNEL-DAPI double staining. After 30 min of fixation with 4% PFA at room temperature, the slices (5 μm) were incubated for 10 min with 0.5% Triton X-100, supplemented with TUNEL reaction mixture, cultured together for 60 min at 37 °C, and observed under a fluorescence microscope (Nikon Eclipse Ti-S, Melville, NY, USA). Six visual fields were randomly selected from each slide to count TUNEL-positive cells and calculate the percentage of TUNEL-positive cells.

### Cell culture and transfection

The primary murine PMVECs (Cell Bank of Type Culture Collection of the Chinese Academy of Sciences, Shanghai, China) were cultured in Dulbecco's modified Eagle's medium (Sigma-Aldrich) containing 10% fetal bovine serum in a 37 °C incubator with 5% CO_2_ [40].

The cells were allocated to 10 groups: control group (no treatment), LPS group [PMVECs were treated with 10 μg/mL LPS (from Escherichia coli O55:B5, Sigma-Aldrich) for 6-h to establish the in vitro model of sepsis-induced ALI], LPS + oe-NC group (PMVECs were treated with LPS for 6-h after 24-h transfection with oe-NC), LPS + oe-H19 group (PMVECs were treated with LPS for 6-h after 24-h transfection with oe-H19), mimic NC group (PMVECs were treated with LPS for 6-h after 24-h transfection with mimic NC), miR-mimic group (PMVECs were treated with LPS for 6-h after 24-h transfection with miR-107 mimic), LPS + oe-H19 + mimic NC group (PMVECs were treated with LPS for 6-h after 24-h co-transfection with oe-H19 and mimic NC), LPS + oe-H19 + miR-mimic group (PMVECs were treated with LPS for 6-h after 24-h co-transfection with oe-H19 and miR-107 mimic), LPS + oe-H19 + si-NC group (PMVECs were treated with LPS for 6-h after 24-h co-transfection with oe-H19 and si-NC), and LPS + oe-H19 + si-TGFBR3 group (PMVECs were treated with LPS for 6-h after 24-h co-transfection with oe-H19 and si-TGFBR3). Plasmid vector overexpressing H19 (oe-H19) and its control oe-NC, miR-107 mimic and its control mimic NC, siRNA of TGFBR3 and their controls (oe-NC, mimic NC, and si-NC) were obtained from GenePharma (Shanghai, China), and introduced into PMVECs using Lipofectamine 2000 (Invitrogen) at the final concentration of 50 nM. The experiment was repeated 3 times for each group.

### Flow cytometry analysis

PMVECs in each group were harvested, washed with PBS 3 times, resuspended in binding buffer, and stained using the Annexin V/FITC kit (BD Bioscience, Franklin Lakes, NJ, USA). PMVECs were cultured with Annexin V/FITC and PI at room temperature for 10–15 min without light exposure. The apoptosis of PMVECs was detected using flow cytometry (Aceabio, San Diego, CA, USA) within 1-h. The early stage of PMVEC apoptosis was detected by Annexin V+ and PI- while the late stage apoptosis was detected by Annexin V+ and PI+. The cells in the early and late stages of apoptosis were counted.

### Reverse transcription-quantitative polymerase chain reaction (RT-qPCR)

Once total RNA was extracted from lung tissues and cells using a TRIzol kit (Invitrogen) [41], it was first necessary to detect RNA concentration and purity using a UV spectrophotometer (UV-1800, Shimadzu, Kyoto, Japan) and measure RNA quantity and integrity using a NanoDrop spectrophotometer (Thermo Fisher Scientific) and an Agilent Bioanalyzer RNA 6000 Nano kit (Agilent Technologies, Beijing, China) [42]. The expression pattern of H19 was tested using the One Step SYBR®PrimeScript®PLUS RT-RNA PCR kit (Takara, Dalian, China). The expression pattern of miR-107 was determined using SYBR GREEN primer and 2 × Power Taq PCR MasterMix with GAPDH and U6 as internal controls. The 2^−ΔΔCt^ method was adopted for data analyses [38]. Table 1 shows the primer sequences.

**Table 1:** Primer sequences

Name of primer	Sequences (5′-3′)
H19	F: CTGAGCTAGGGTTGGAGAGG
	R: TTAGAAGGTCAGTGCAGCGA
miR-107	F: AGCAGCATTGTACAGGGCTATGA
	R: GCTCTAGAATCGGTGAGCACTG
TGFBR3	F: CCTAAGTGTGTGCCTCCTGA
	R: CAATGCCCATCACGGTTAGG
U6	F: ATGGCGGACGACGTAGATCAGCA
	R: TCAGCCAACTCTCAATGGAGGGGC
GAPDH	F: ATGGTGAAGGTCGGTGTGAACGGA
	R: TTACTCCTTGGAGGCCATGTAGGC

*miR* microRNA; *TGFBR3* transforming growth factor type III receptor; *GAPDH* glyceraldehyde-3-phosphate dehydrogenase

### Enzyme-linked immunosorbent assay (ELISA)

The lung tissues and PMVECs were lysed with radioimmunoprecipitation assay (RIPA) lysis buffer (Solarbio, Beijing, China) containing protease inhibitor, and centrifuged (14,000 × g, 5 min) to collect the supernatant. The levels of TNF-α (70-EK182HS-96), interleukin (IL)-1β (70-EK201B/3-96), IL-6 (70-EK206/3-96), and IL-10 (70-EK210/4-96) in the BALF, lung tissue homogenate or supernatant were determined using ELISA kits (Lianke Biotechnology, Hangzhou, China) following the standard procedures.

### Dual-luciferase reporter assay

The binding site between lncRNA H19 and miR-107, and that between miR-107 and TGFBR3 were predicted on the Starbase database (http://starbase.sysu.edu.cn/). For the construction of wild-type plasmids pmirGLO-H19-WT and pmirGLO-TGFBR3-WT and corresponding mutant plasmids pmirGLO-H19-MUT and pmirGLO-TGFBR3-MUT, the complementary and mutant sequences of miR-107, lncRNA H19 and TGFBR3 were amplified and cloned to pmir-GLO luciferase reporter vectors (GenePharma). These plasmids were co-delivered into PMVECs with mimic NC or miR-107 mimic using Lipofectamine 2000 based on the provided instructions. Luciferase activity was detected after 48-h using dual-luciferase detection kits (Promega, Madison, WI, USA).

### Western blot

The lung tissues or PMVECs were lysed with RIPA lysis buffer (Solarbio) containing protease inhibitor. Protein concentration was detected using a BCA kit (Beyotime, Shanghai, China). The protein sample was separated in 10% sodium dodecyl sulfate–polyacrylamide gel electrophoresis [37], and moved to the polyvinylidene fluoride membranes (Millipore, Billerica, MA, USA). To block non-specific binding, the membranes were placed in Tris-buffered saline-Tween-20 with 5% skim milk on the shaking table for 1-h at room temperature. The membranes were added with primary antibodies against Bax (ab0261, 1:2000, Beyotime), Bcl-2 (ab182858, 1:800, Abcam, Cambridge, MA, USA) and TGFBR3 (ab166705, 1:800, Abcam) for overnight incubation at 4 °C. The membranes were washed and then incubated with HRP-labeled goat anti-rabbit secondary antibody IgG (ab48386, 1:2000, Abcam) at room temperature for 1-h [42], visualized with enhanced chemiluminescence working solution (EMD Millipore, Billerica, MA, USA), and images were captured. The protein band density was detected using Image J software 1.48 (NIH, Bethesda, MD, USA) with β-actin as an internal control.

### RNA immunoprecipitation (RIP) assay

After lysing PMVECs with RIPA lysis buffer (Solarbio), PMVECs were centrifuged at 1200 ×g for 30 min to collect the supernatant. A small portion of the supernatant served as Input and the remaining supernatant was probed with anti-Ago2 or anti-IgG (Sigma-Aldrich) overnight at 4 °C and then added with 40 μL protein A magnetic beads to get the immunoprecipitation complex. The GenElute™ Total RNA purification kit (Sigma-Aldrich) was used to extract total RNA. RT-qPCR was run to analyze the relative RNA enrichment of H19 and miR-107.

### Statistical analysis

Data analysis was performed using SPSS21.0 statistical software (IBM Corp., Armonk, NY, USA). Measurement data were reported as mean ± standard deviation. Comparisons between the two groups were made using the independent sample *t* test and comparisons among multi-groups were made using one-way or two-way analysis of variance (ANOVA). Post-hoc test was conducted using Tukey's test. The value of *p* < 0.05 was of great statistical significance.

## Results

### Overexpression of lncRNA H19 attenuates sepsis-induced ALI

**Fig. 1:**
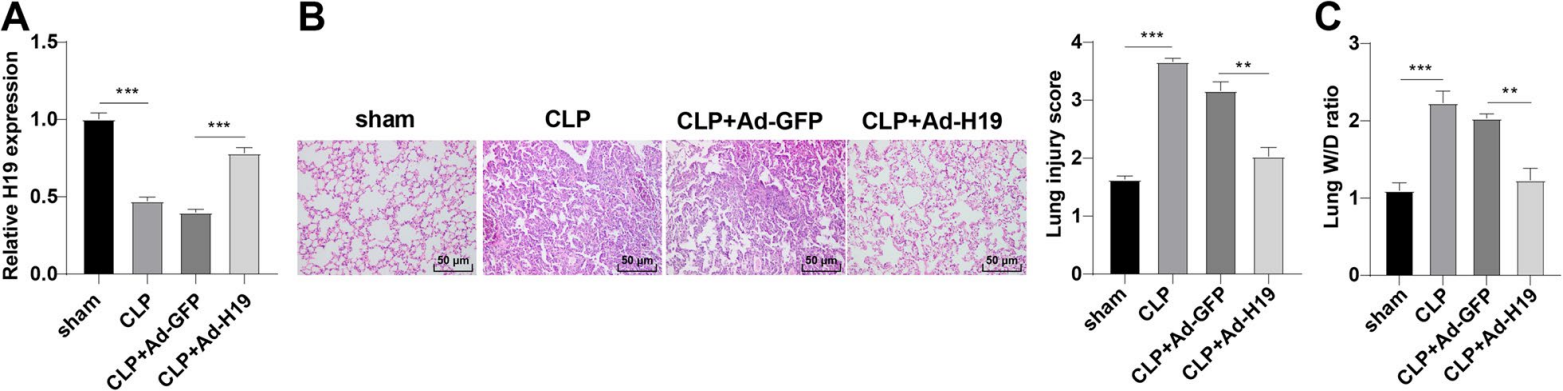
Overexpression of lncRNA H19 attenuates sepsis-induced ALI. The C57BL/6 mouse model of sepsis-induced ALI was established via CLP. **A** The relative expression pattern of H19 in lung tissues of mice was determined by RT-qPCR; **B** the morphological changes of the lung tissues of sham-operated mice and CLP-operated mice were analyzed by H&E staining, and lung injury was evaluated by lung injury scores; **C** the pulmonary edema was assessed by analyzing the lung W/D ratio. N = 6. Data were presented as mean ± standard deviation. Comparisons among multi-groups were made using one-way ANOVA and Tukey’s multiple comparisons test. **p < 0.01, *** *p* < 0.001

Sepsis is defined as a SIRS leading to acute injury of multiple organs, among which the lung is the first to be impacted [10, 43, 44]. To investigate the function and mechanism of H19 in sepsis-induced ALI, the C57BL/6 mouse model of sepsis was established by CLP. Mice were intravenously injected with adenovirus vector Ad-H19 prior to CLP to overexpress H19 in vivo, and RT-qPCR revealed that the CLP group presented decreased H19 expression relative to the sham group, while the CLP + Ad-H19 group manifested increased H19 expression relative to the CLP + Ad-GFP group (all *p* < 0.01, Fig. 1A). Mice were subsequently euthanized and lung tissues were immediately collected, followed by H&E staining to observe pathological changes, which showed normal alveolar structure in sham-operated mice and damaged alveolar structure accompanied by congestion and collapse, interstitial and alveolar cell infiltration, and thickened alveolar wall with visible red blood cells was observed in CLP-operated mice, suggesting that CLP induced severe damage to the lung tissues, whereas the pathological changes of lung tissues were relieved and alveolar structure was more complete in mice in the CLP + Ad-H19 group; the lung injury scores of the CLP group were higher than those of the sham group, whilst the lung injury scores of the CLP + Ad-H19 group were lower than those of the CLP + Ad-GFP group (all *p* < 0.01, Fig. 1B). The lung W/D ratio of the CLP-operated mice was significantly elevated compared to that of the sham-operated mice, and H19 overexpression reduced the lung W/D ratio (all *p* < 0.01, Fig. 1C), indicating that severe pulmonary edema occurred after CLP, and H19 overexpression could mitigate pulmonary edema. These results pointed out that lncRNA H19 was poorly-expressed in lung tissues of mice with sepsis-induced ALI, and H19 overexpression could ameliorate sepsis-induced ALI.

### Overexpression of H19 suppresses apoptosis and inflammation in the lungs of mice with sepsis-induced ALI

**Fig. 2:**
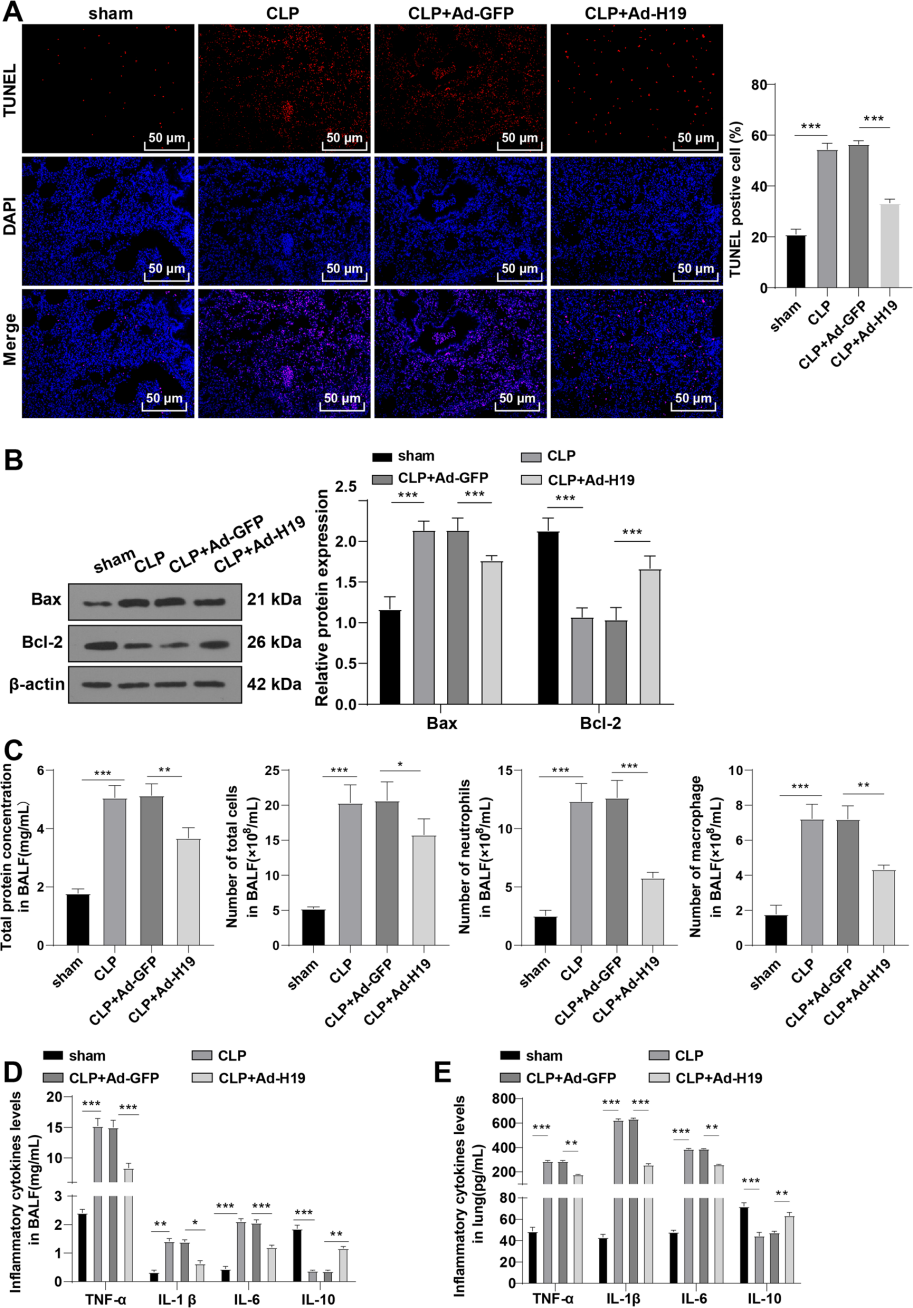
Overexpression of H19 inhibits apoptosis and inflammation in lungs of mice with sepsis-induced ALI. **A** The lung tissue injury was assessed by TUNEL staining, N = 6; **B** the levels of Bax and Bcl-2 were measured by Western blot, N = 6; **C** the total protein concentration was measured and the number of total cells, neutrophils and macrophages in BALF of mice was counted, N = 12; **D, E** the levels of inflammatory cytokines (TNF-α, IL-1β, IL-6, and IL-10) in BALF (N = 12) and lung tissues (N = 6) of mice were determined by ELISA. Data were presented as mean ± standard deviation. Comparisons among multi-groups were made using one-way ANOVA and Tukey’s multiple comparisons test. *p < 0.05, **p < 0.01, ***p < 0.001

TUNEL staining showed an increased number of TUNEL-positive cells in CLP-operated mice relative to sham-operated mice and overexpression of H19 significantly decreased the number of TUNEL-positive cells (all *p* < 0.01, Fig. 2A). Additionally, Western blot showed increased levels of Bax and decreased levels of Bcl-2 in CLP-operated mice, which was averted after overexpression of H19 (all *p* < 0.01, Fig. 2B) (Additional file 1). Furthermore, the CLP-operated mice had an elevated total protein concentration and an augmented number of total cells, neutrophils, and macrophages in the BALF relative to the sham-operated mice, whereas H19 overexpression invalidated these changes (all *p* < 0.01, Fig. 2C). ELISA showed elevated levels of TNF-α, IL-1β, and IL-6 and decreased IL-10 levels in BALF and lung tissues of CLP-operated mice, while overexpression of H19 inhibited the levels of pro-inflammatory cytokines and enhanced the level of anti-inflammatory cytokine (all *p* < 0.01, Fig. 2D, E). These results together indicated that overexpression of H19 inhibited apoptosis and inflammation in the lungs of mice with sepsis-induced ALI.

### Overexpression of H19 suppresses apoptosis of PMVECs and release of inflammatory cytokines induced by LPS

**Fig. 3:**
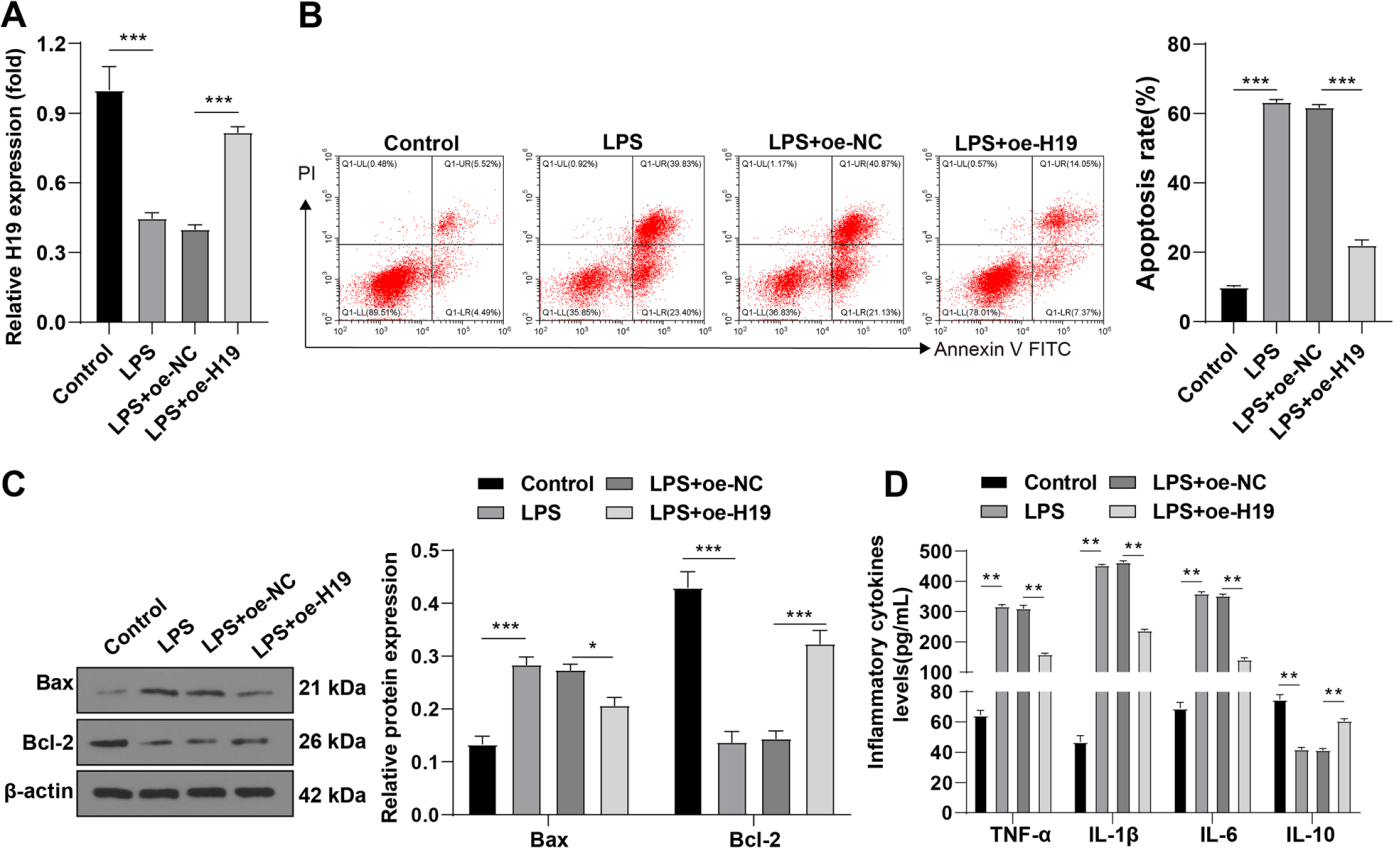
Overexpression of H19 suppresses apoptosis of PMVECs and release of pro-inflammatory cytokines induced by LPS. The in vitro model of sepsis-induced ALI was established using LPS-induced PMVECs and H19 was overexpressed via transfection. **A** The relative expression pattern of H19 was determined by RT-qPCR; **B** the apoptotic rate of LPS-induced PMVECs was estimated by flow cytometry; **C** the relative expression levels of Bax and Bcl-2 were measured by Western blot; **D** the levels of TNF-α, IL-1β, IL-6 and IL-10 in PMVECs were measured by ELISA. Cell experiments were repeated 3 times. Data were presented as mean ± standard deviation. Comparisons among multi-groups were made using one-way ANOVA and Tukey’s multiple comparisons test. **p < 0.01, ***p < 0.001

Subsequently, an in vitro model of sepsis-induced ALI was established using LPS-induced PMVECs, and H19 was overexpressed in LPS-induced PMVECs via transfection to explore the specific in vitro mechanism of H19 in cell apoptosis and secretion of inflammatory cytokines. First of all, H19 expression in PMVECs was measured by RT-qPCR, which showed that LPS treatment reduced H19 expression in PMVECs, and the expression level of H19 was increased in the LPS + oe-H19 group relative to the LPS + oe-NC group (all *p* < 0.01, Fig. 3A). Subsequently, flow cytometry showed an increased apoptotic rate of LPS-induced PMVECs, and overexpression of H19 reduced the apoptotic rate (all *p* < 0.01, Fig. 3B). Simultaneously, LPS remarkably induced the release of Bax and inhibited the relative level of Bcl-2 in PMVECs while overexpression of H19 reduced Bax level and raised Bcl-2 level (all *p* < 0.01, Fig. 3C) (Additional file 2). According to the result of ELISA, levels of TNF-α, IL-1β, and IL-6 were raised and IL-10 level was decreased in the LPS group while overexpression of H19 reduced levels of TNF-α, IL-1β, and IL-6 and increased IL-10 level (all *p* < 0.01, Fig. 3D). Briefly, overexpression of H19 inhibited LPS-induced apoptosis of PMVECs and the release of pro-inflammatory cytokines.

### LncRNA H19 targets miR-107

**Fig. 4:**
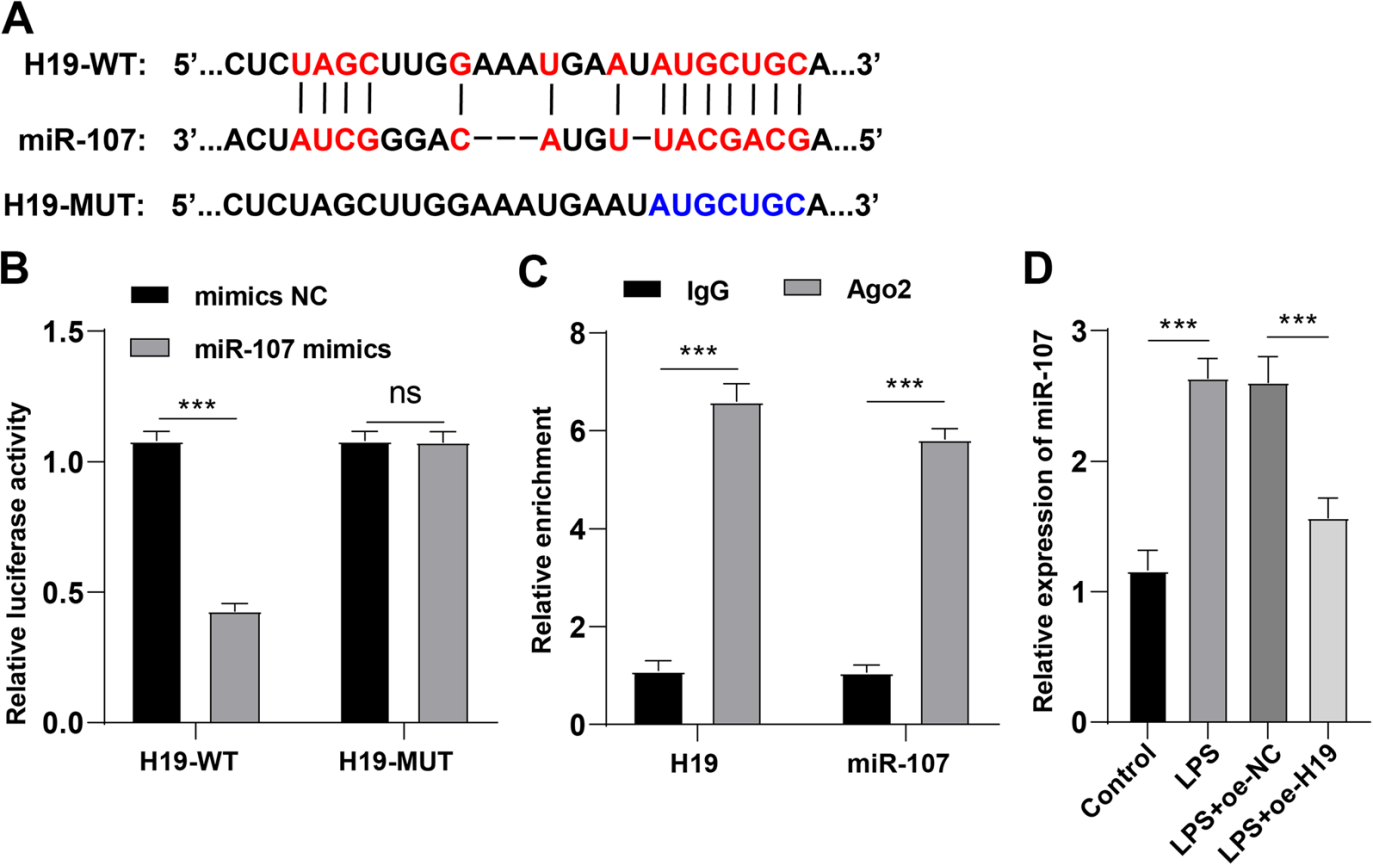
LncRNA H19 targets miR-107. **A** A putative binding site of H19 and miR-107 was predicted on the Starbase database; **B** the target relationship between H19 and miR-107 was verified by dual-luciferase reporter assay; **C** the cross-linking between H19 and miR-107 was analyzed by RIP assay; **D** the effect of H19 overexpression on the relative expression pattern of miR-107 was determined by RT-qPCR. Cell experiments were repeated 3 times. Data were presented as mean ± standard deviation. Comparisons among multi-groups were made using one-way ANOVA and Tukey’s multiple comparisons test. **p < 0.01, ***p < 0.001

H19 plays a regulatory role in sepsis through the ceRNA mechanism [42]. We then turned to the downstream regulatory mechanism of lncRNA H19. miR-107 is highly-expressed in the whole blood of mice with LPS-induced sepsis [29], and miR-107 could induce TNF-α secretion in endothelial cells and thus result in tubular cell injury [28]. Based on the prediction of the Starbase database, there is a binding site between miR-107 and H19 (Fig. 4A). Dual-luciferase reporter assay exhibited lower luciferase activity in PMVECs co-transfected with miR-107 mimic and H19-WT than PMVECs co-transfected with mimic NC and H19-WT (*p* < 0.01), while no obvious change in luciferase activity was observed in PMVECs transfected with H19-MUT (*p* > 0.05, Fig. 4B). As a central part of the RNA-induced silencing complex with endonuclease activity, Ago2 could potentiate miRNA maturation and modulate miRNA biosynthesis and function and hence inhibit the expression levels of target genes [45]. IgG was used as a negative control. RIP assay showed enrichment of H19 and miR-107 in the immunoprecipitation of Ago2, which proved the interaction between H19 and miR-107 (Fig. 4C). In addition, RT-qPCR showed that LPS treatment elevated miR-107 expression while overexpression of H19 greatly reduced miR-107 expression (all *p* < 0.01, Fig. 4D). Overall, H19 targeted miR-107.

### Overexpression of miR-107 partially annuls the inhibitory effect of H19 overexpression on LPS-induced apoptosis of PMVECs

**Fig. 5:**
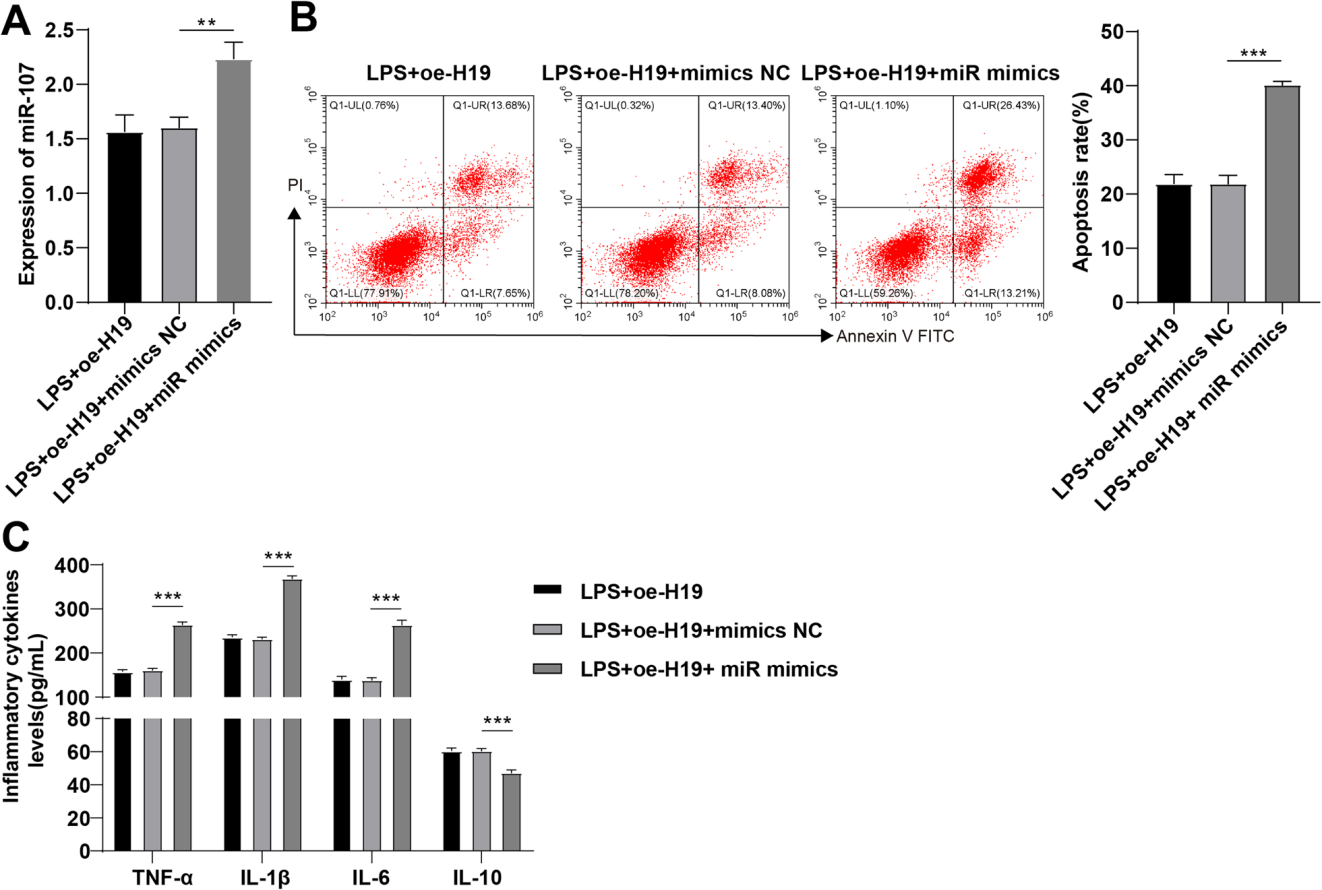
Overexpression of miR-107 partially annuls the inhibitory effect of H19 overexpression on LPS-induced apoptosis of PMVECs. **A** the expression pattern of miR-107 in PMVECs was determined by RT-qPCR; **B** the apoptotic rate of LPS-induced PMVECs was estimated by flow cytometry; **C** the levels of TNF-α, IL-1β, IL-6, and IL-10 in PMVECs were measured by ELISA. Cell experiments were repeated 3 times. Data were presented as mean ± standard deviation. Comparisons among multi-groups were made using one-way ANOVA and Tukey’s multiple comparisons test. **p < 0.01, ***p < 0.001

To further investigate whether H19 could regulate LPS-induced apoptosis of PMVECs as the ceRNA of miR-107, miR-107 overexpression was induced by transfection in H19-overexpressed PMVECs. According to RT-qPCR, miR-107 was upregulated in the miR-107 mimic group relative to the LPS + oe-H19 + mimic NC group (Fig. 5A). Flow cytometry and ELISA showed increased cell apoptotic rate and elevated levels of TNF-α, IL-1β, and IL-6 and decreased IL-10 level in the LPS + oe-H19 + miR mimic group relative to the LPS + oe-H19 + mimic NC group (all *p* < 0.01, Fig. 5B, C). These results in this part indicated that overexpression of miR-107 averted the inhibitory function of H19 overexpression on LPS-induced apoptosis of PMVECs and release of pro-inflammatory cytokines.

### miR-107 directly targets TGFBR3

**Fig. 6:**
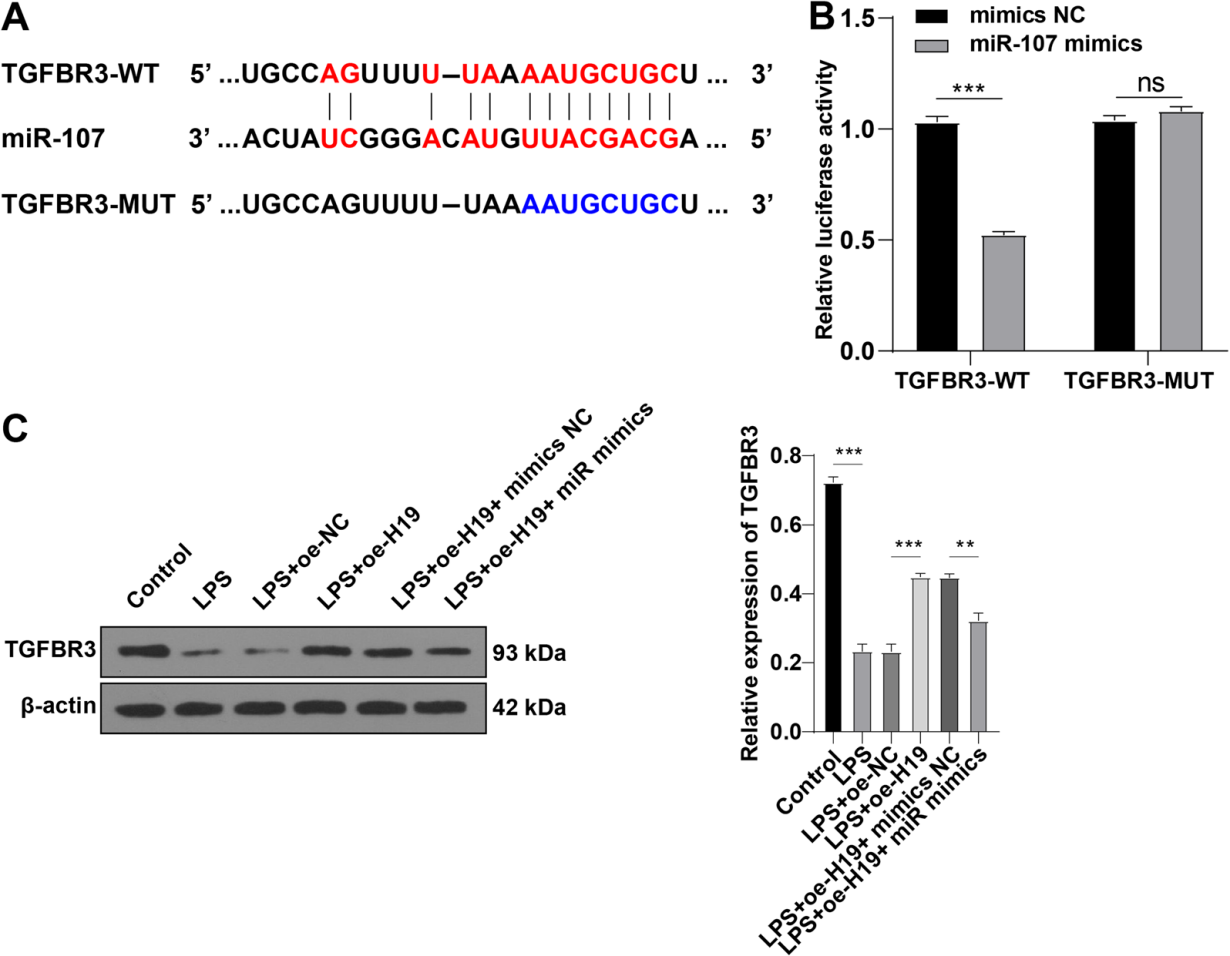
miR-107 targets TGFBR3. **A** The complementary relationship between TGFBR3 and miR-107 was predicted on the Starbase database; **B**: the relative luciferase activity was detected by dual-luciferase reporter assay; **C**: the expression level of TGFBR3 was determined by Western blot. Cell experiments were repeated 3 times. Data were presented as mean ± standard deviation. Comparisons among multi-groups were made using one-way ANOVA and Tukey's multiple comparisons test. **p < 0.01, ***p < 0.001, ns *p* > 0.05

Furthermore, we explored the downstream regulatory mechanism of the H19/miR-107 axis in sepsis-induced ALI. TGFBR3 is a pivotal gene that is downregulated in sepsis [32], and TGFBR3 participates in the regulation of inflammation in ALI [31, 33]. According to the Starbase database, the 5’ terminal region of miR-107 was complementary to the 3’ UTR of TGFBR3 (Fig. 6A). Dual-luciferase reporter assay showed lower luciferase activity in PMVECs co-transfected with miR-107 mimic and TGFBR3-WT than PMVECs co-transfected with mimic NC and TGFBR3-WT (*p* < 0.01) while no obvious change in luciferase activity was observed in PMVECs transfected with TGFBR3-MUT (*p* > 0.05, Fig. 6B). Western blot showed that TGFBR3 level was decreased after LPS treatment and elevated after overexpression of H19 (all *p* < 0.01), while overexpression of miR-107 reduced TGFBR3 level (*p* < 0.05, Fig. 6C) (Additional file 3). Taken together, these results suggested that miR-107 targeted TGFBR3.

### Silencing TGFBR3 partially inverts the repressive effect of H19 overexpression on LPS-induced apoptosis of PMVECs

**Fig. 7:**
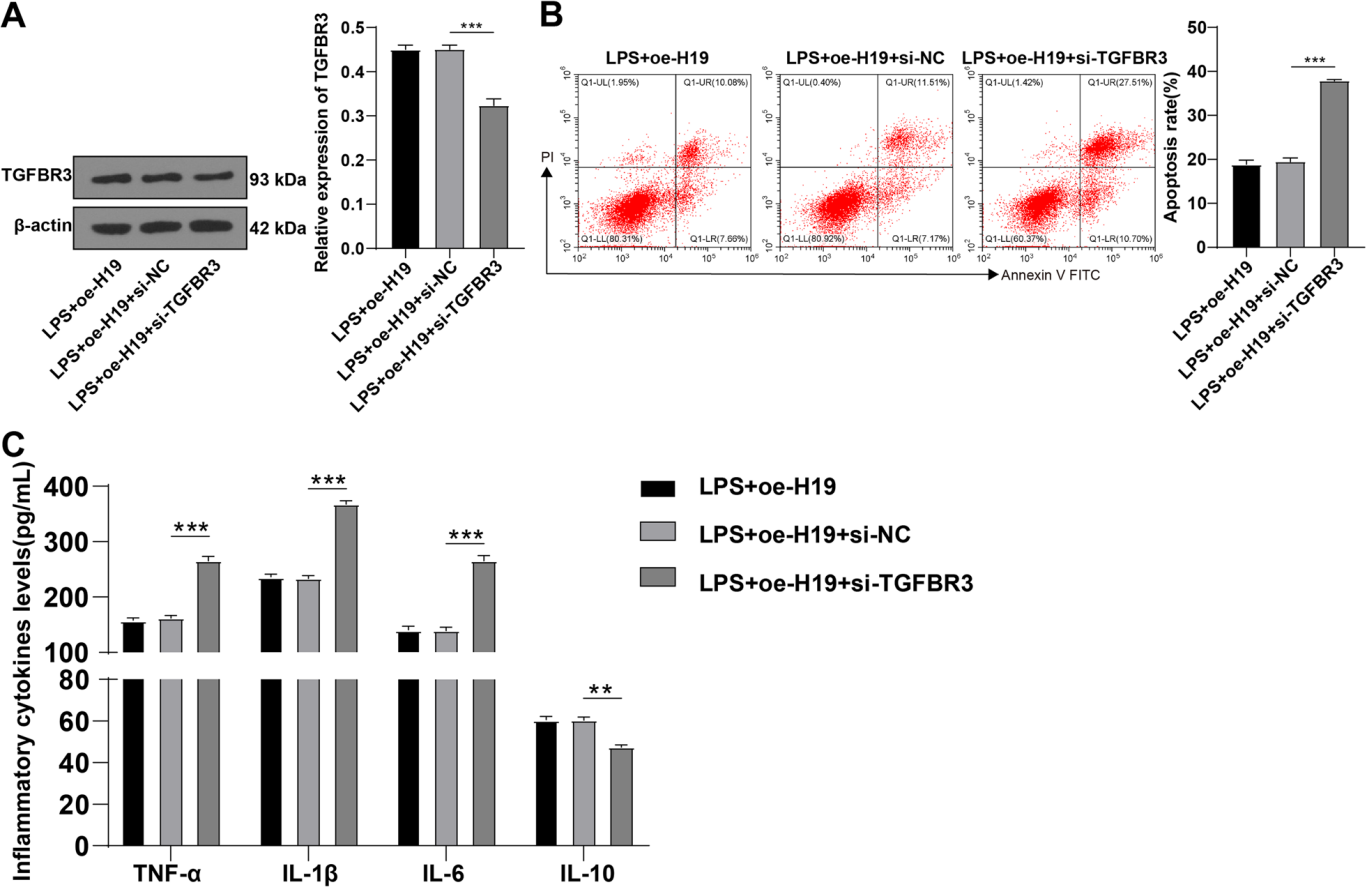
Silencing TGFBR3 partially inverts the inhibitory effect of H19 overexpression on LPS-induced apoptosis of PMVECs. **A** the relative expression level of TGFBR3 was determined by Western blot; **B** the apoptotic rate was estimated by flow cytometry; **C**: the levels of TNF-α, IL-1β, IL-6, and IL-10 in PMVECs were measured by ELISA. Cell experiments were repeated 3 times. Data were presented as mean ± standard deviation. Comparisons among multi-groups were made using one-way ANOVA and Tukey’s multiple comparisons test. **p < 0.01, ***p < 0.001

Next, we silenced TGFBR3 and overexpressed H19 by delivering oe-H19 and si-TGFBR3 into PMVECs. Western blot showed decreased TGFBR3 expression level in the LPS + oe-H19 + si-TGFBR3 group relative to the LPS + oe-H19 + si-NC group (*p* < 0.01, Fig. 7A) (Additional file 4). Flow cytometry and ELISA manifested that the LPS + oe-H19 + si-TGFBR3 group had increased apoptotic rate and levels of TNF-α, IL-1β, and IL-6 and decreased IL-10 levels relative to the LPS + oe-H19 + si-NC group (all *p* < 0.01, Fig. 7B, C). To sum up, silencing TGFBR3 partially abolished the inhibitory function of H19 overexpression on LPS-induced PMVEC apoptosis and the release of pro-inflammatory cytokines.

## Discussion

Sepsis-induced ALI, or more accurately, acute respiratory distress syndrome is a significant contributor to mortality and morbidity in septic patients [46, 47]. Several reports have shown that lncRNA H19 is capable of protecting against both ALI [48] and sepsis [23]. The primary finding in this research was that lncRNA H19 inhibited inflammation in sepsis-induced ALI by regulating the miR-107/TGFBR3 axis. We found that lncRNA H19 was poorly-expressed in the mouse model and cell model of sepsis-induced ALI, while overexpression of H19 suppressed apoptosis and inflammation in sepsis-induced ALI in vivo and in vitro, in which the miR-107/TGFBR3 axis was involved.

As mentioned in the literature, lncRNA H19 is downregulated in the CLP mouse model and cell model of LPS-stimulated cell injury, while upregulation of H19 can ameliorate lung injury in mice with sepsis [49]. In consistency with the aforementioned study, poorly-expressed H19 was observed in the lung tissues of mice with sepsis-induced ALI and LPS-treated PMVECs. Meanwhile, the CLP-operated mice showed intact alveolar structure and the lung tissue lesion was mitigated and lung injury scores and lung W/D ratio were lowered after H19 overexpression. Inflammation and abnormal apoptosis are involved in the pathophysiological mechanism of sepsis-induced ALI [50]. The CLP-operated mice in our study showed decreased number of TUNEL-positive cells after H19 overexpression. Meanwhile, apoptosis of LPS-treated PMVECs was reduced after H19 overexpression. Anti-apoptotic protein Bcl-2 and pro-apoptotic protein Bax play opposite roles in apoptosis [51]. The increased Bax level and decreased Bcl-2 level in the CLP-operated mice and LPS-treated PMVECs were inverted after H19 overexpression. The influx of neutrophils is another indicator of significant pulmonary injury [52]. Macrophages are vital immune cells in the lung tissues [53]. H19 overexpression reduced total protein concentration and the number of total cells, neutrophils, and macrophages in BALF of mice with sepsis-induced ALI. Additionally, levels of pro-inflammatory cytokines TNF-α, IL-1β, and IL-6 were decreased and the level of anti-inflammatory cytokine IL-10 was increased in BALF and lung tissues after H19 overexpression. Similar results were obtained by in vitro assay. The knockdown of lncRNA H19 induces apoptosis of vascular smooth muscle cells stimulated by ox-LDL [54]. On the contrary, overexpression of H19 alleviates inflammation and apoptosis of cardiomyocytes in diabetic rats [55]. Taken together, overexpression of H19 inhibited the production of pro-inflammatory cytokines and apoptosis in sepsis-induced ALI.

Considering that H19 negatively regulates miR-874 expression in LPS sepsis [23], we searched the downstream miRNAs of H19 and identified miR-107. A prior study has noted the upregulation of miR-107 in the whole blood of mice exposed to LPS [29]. The target relationship between miR-107 and H19 was verified by dual-luciferase assay and RIP assay. It can thus be suggested that H19 targeted miR-107. Furthermore, our results manifested that miR-107 overexpression increased the apoptotic rate and levels of TNF-α, IL-1β, and IL-6 and reduced level of IL-10 in PMVECs. Overexpression of miR-107 is associated with the secretion of pro-inflammatory cytokines in sepsis-induced kidney injury [28]. miR-107 facilitates apoptosis of preadipocytes mediated by endoplasmic reticulum stress [56]. In short, H19 could repress the apoptosis of PMVECs and secretion of pro-inflammatory cytokines in vitro by targeting miR-107.

In reviewing the literature, TGFBR3 was found downregulated in sepsis [32]. miR-15b-5p is negatively correlated with TGFBR3 in H_2_S-induced necroptosis and inflammation [33]. In this study, we found a complementary relationship between the 5′ terminal region of miR-107 and the 3′ UTR of TGFBR3, and H19 overexpression increased the expression level of TGFBR3 in PMVECs while miR-107 overexpression downregulated TGFBR3. These results highlighted that miR-107 targeted TGFBR3. TGFBR3 can protect cardiac fibroblasts from apoptosis [57]. After silencing TGFBR3, the apoptotic rate was raised and levels of TNF-α, IL-1β, and IL-6 were elevated, whereas IL-10 level was declined. In IL-1β-stimulated osteoarthritis, the inhibitory effect of SNHG5 on the apoptosis of chondrocytes and inflammation is offset by silencing TGFBR3 [58]. To summarize, lncRNA H19 could reduce apoptosis of LPS-induced PMVECs and release of pro-inflammatory cytokines in vitro through the miR-107/TGFBR3 axis.

Despite these promising results, questions remain. One major drawback of the study is the lack of sufficient investigation into the regulatory mechanism of lncRNA H19 in inflammation in sepsis-induced ALI, for example, through modulating several signaling pathways downstream of TGFBR3, which we plan to include in future studies. In addition, due to restrictions on experimental periods and funds, we only observed the changes of cytokines in lung tissues and BALF, while the expression levels of serum cytokines and other organ injury markers were left unmeasured. This is an important issue for future research. Moreover, there are still many unanswered questions about whether lncRNA H19 has any impact on tissue regeneration or the effects of coagulation, which shall be the research emphasis in the future. Besides, the role of TGFBR3 was superficially explored in this study. More efforts should be devoted to discussing the downstream signaling pathways affected by TGFBR3. The future directions comprise the specific mechanisms of lncRNA H19 in attenuating sepsis-induced ALI and whether H19 could be the breakthrough point in the management of sepsis, with the expectation to complete the molecular network of H19 in sepsis-triggered ALI and provide a more systematic and reliable reference for sepsis management. In conclusion, the present study highlighted that lncRNA H19 ameliorated apoptosis and inflammation in sepsis-induced ALI through the miR-107/TGFBR3 axis.

## Supplementary Information

**Additional file 1**. Expression levels of Bax and Bcl-2 were determined by Western blot.

**Additional file 2**. Relative expression levels of Bax and Bcl-2 were determined by Western blot.

**Additional file 3**. Expression level of TGFBR3 were determined by Western blot.

**Additional file 4**. Relative expression level of TGFBR3 were determined by Western blot.

## Data Availability

The data that support the findings of this study are available from the corresponding author upon reasonable request.
