# Supplementary figures and images for: LncRNA H19 alleviates sepsis-induced acute lung injury by regulating the miR-107/TGFBR3 axis
Source: BMC Pulm Med. 2022 Sep 30;22:371. doi: 10.1186/s12890-022-02091-y (PMC9524034; doi:10.1186/s12890-022-02091-y)

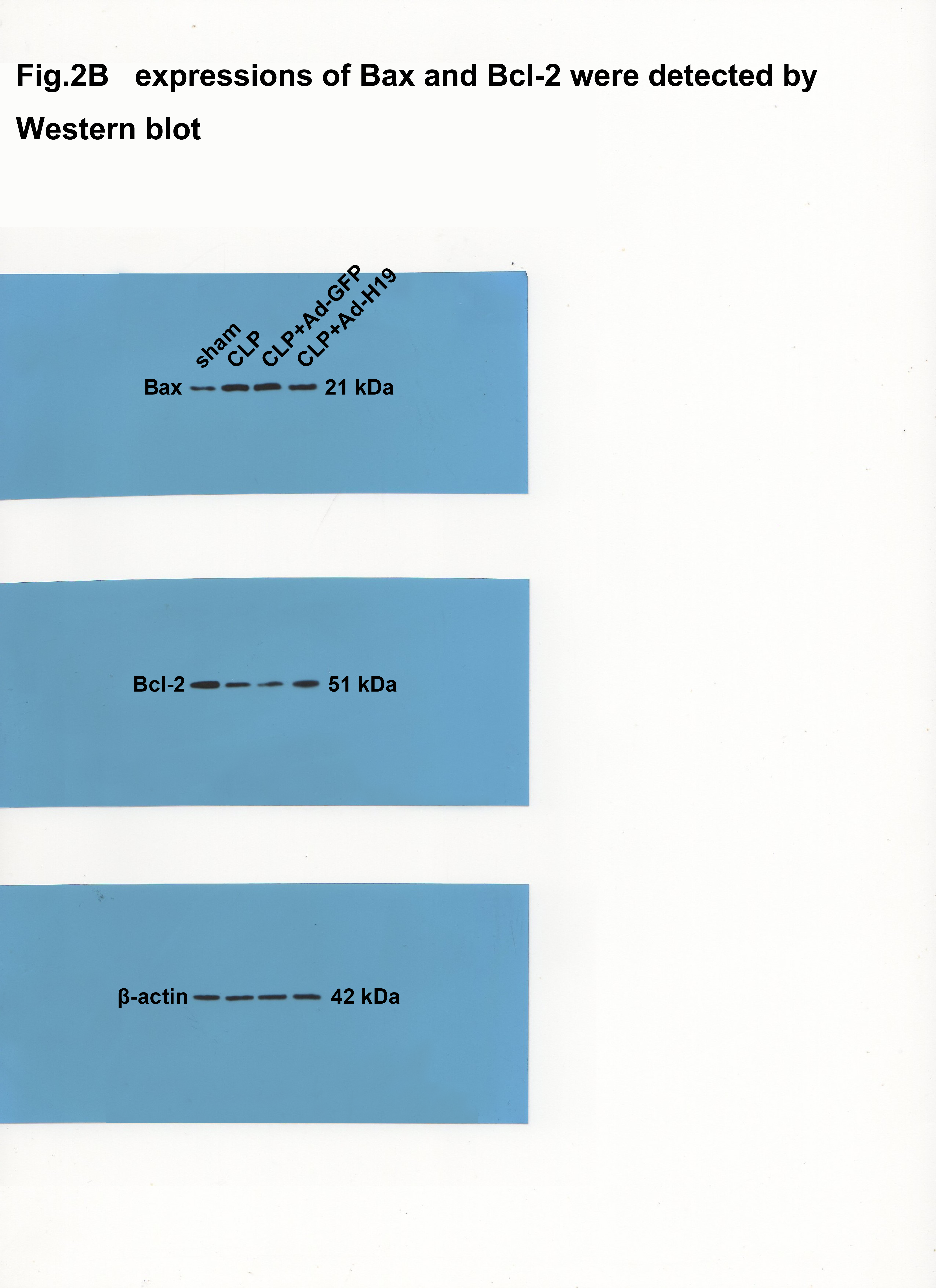

Supplement: Supplementary file 1 — Additional file 1. Expression levels of Bax and Bcl-2 were determined by Western blot. [file 12890_2022_2091_MOESM1_ESM.tif]

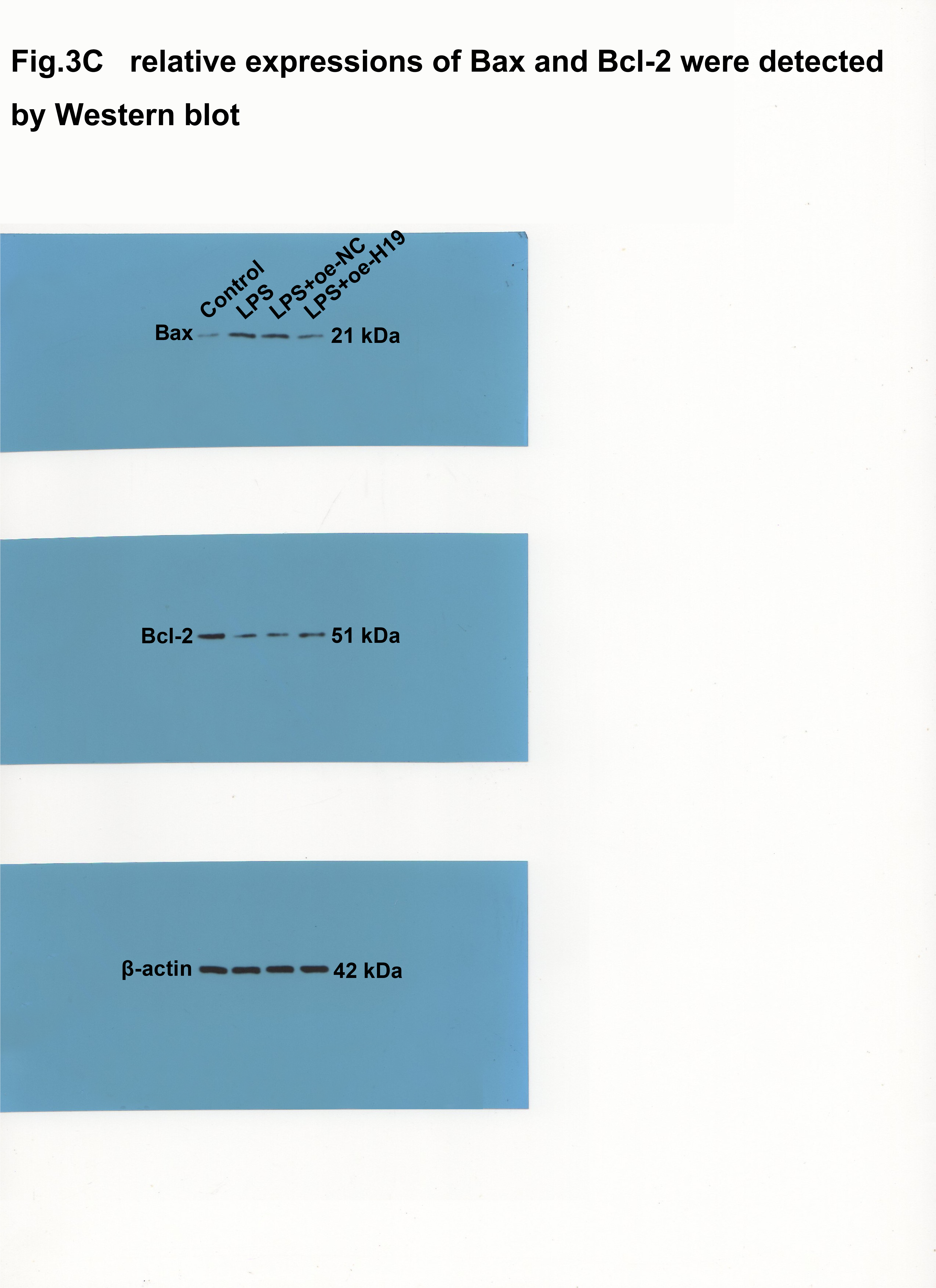

Supplement: Supplementary file 2 — Additional file 2. Relative expression levels of Bax and Bcl-2 were determined by Western blot. [file 12890_2022_2091_MOESM2_ESM.tif]

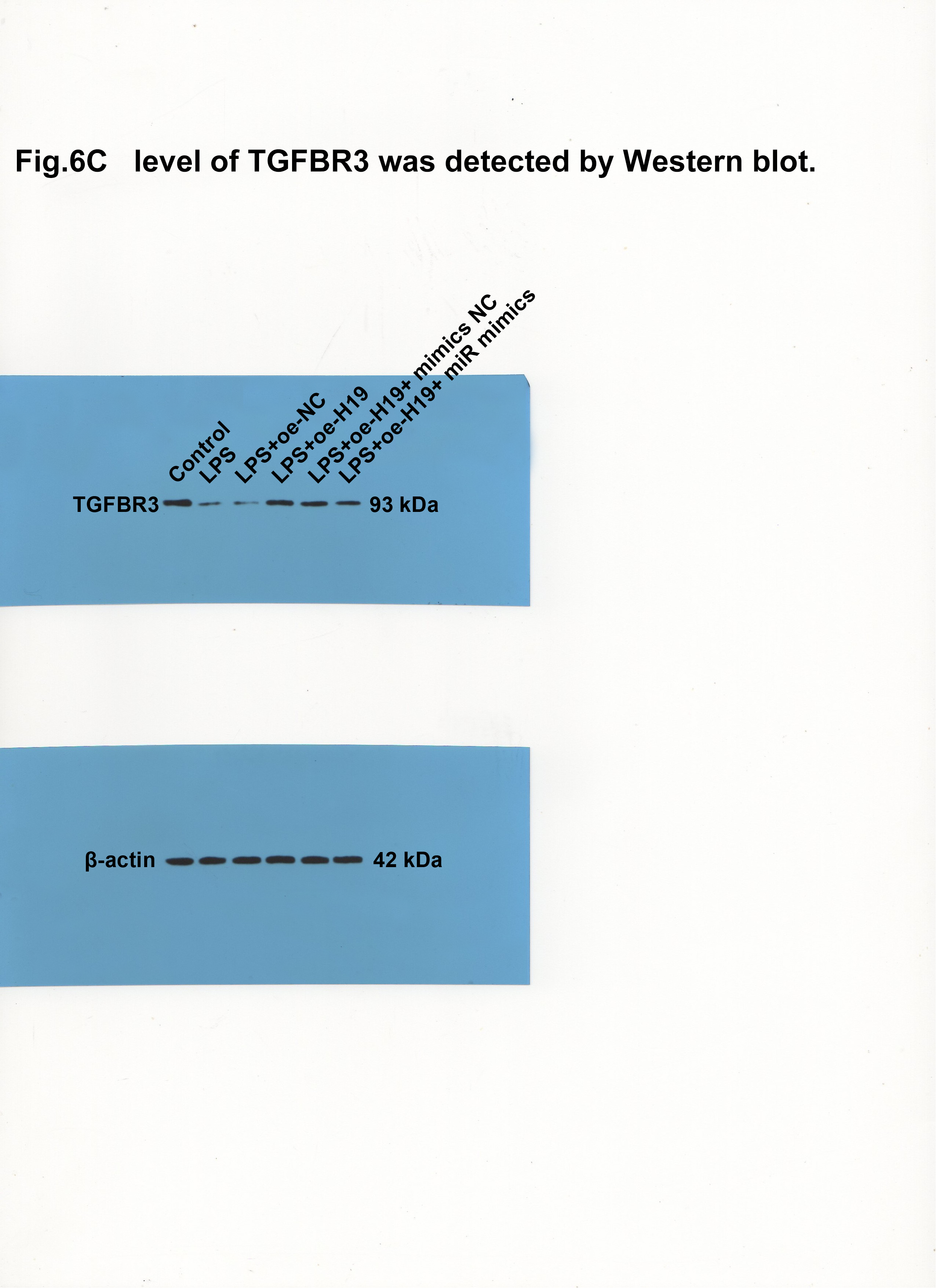

Supplement: Supplementary file 3 — Additional file 3. Expression level of TGFBR3 were determined by Western blot. [file 12890_2022_2091_MOESM3_ESM.tif]

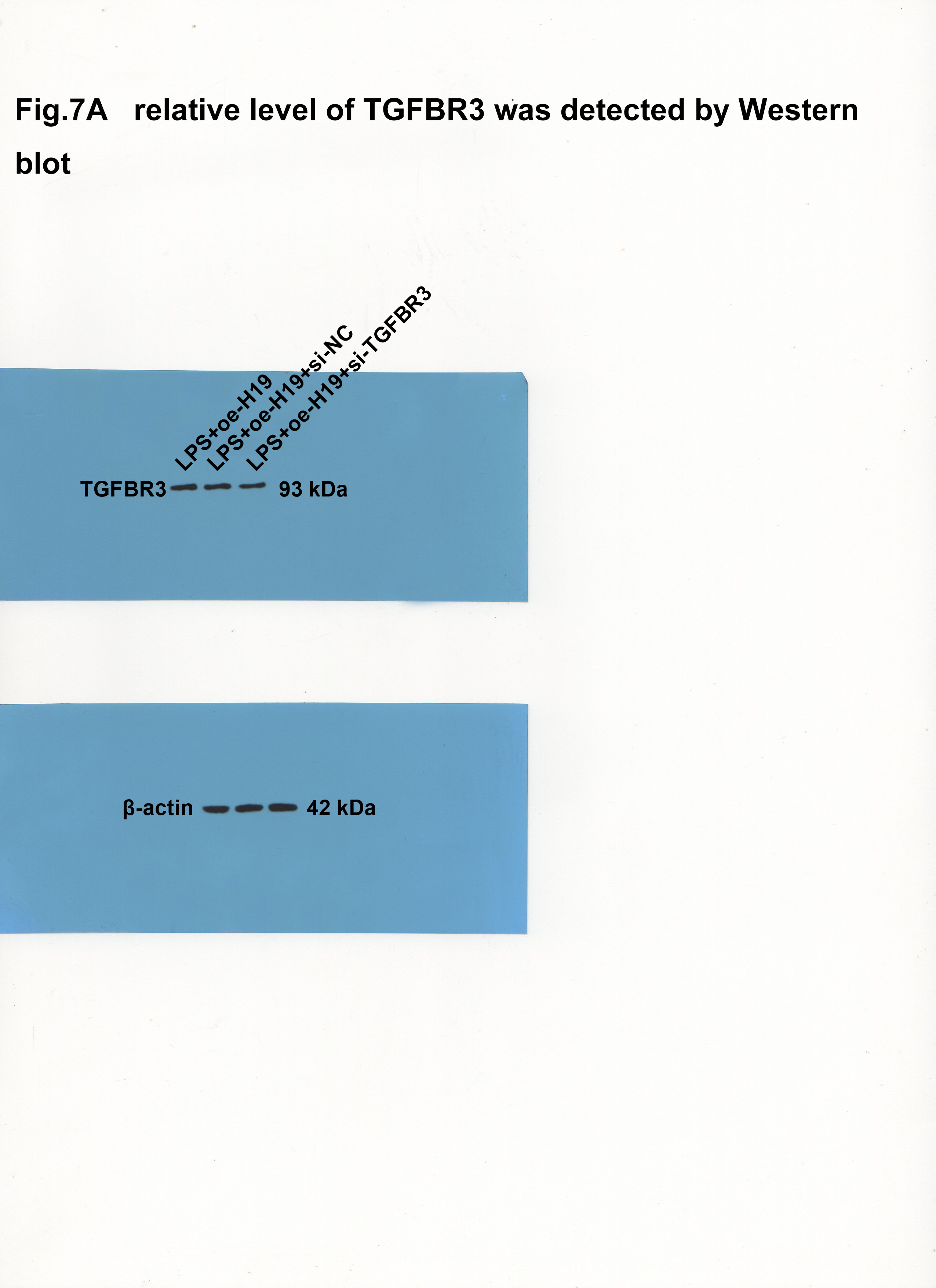

Supplement: Supplementary file 4 — Additional file 4. Relative expression level of TGFBR3 were determined by Western blot. [file 12890_2022_2091_MOESM4_ESM.tif]
